# Supplementary figures and images for: FOXO1 transcription factor modulates airway epithelial responses to viral infection
Source: PLoS One. 2026 Apr 3;21(4):e0345169. doi: 10.1371/journal.pone.0345169 (PMC13048417; doi:10.1371/journal.pone.0345169)

A

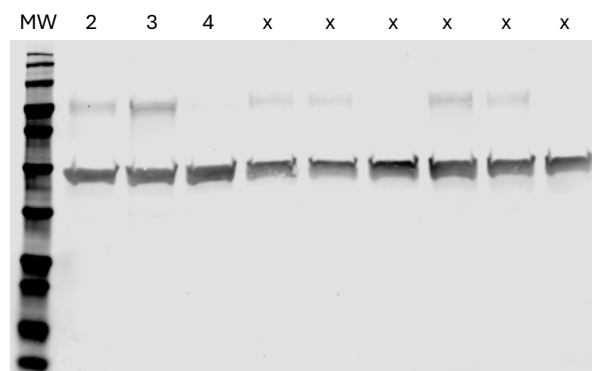

D

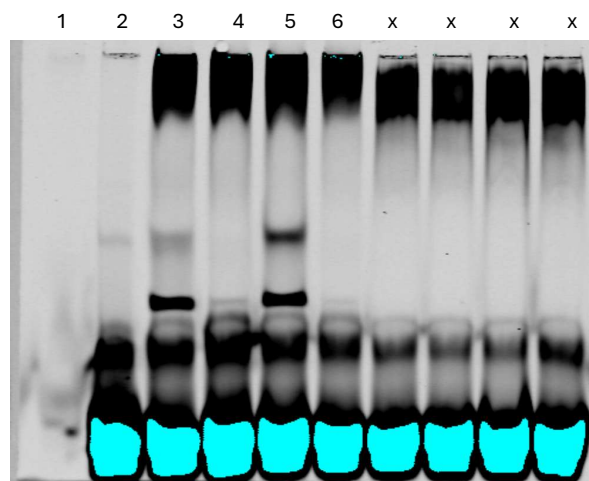

B

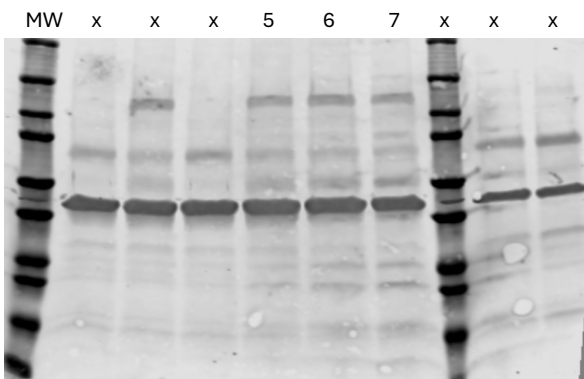

E

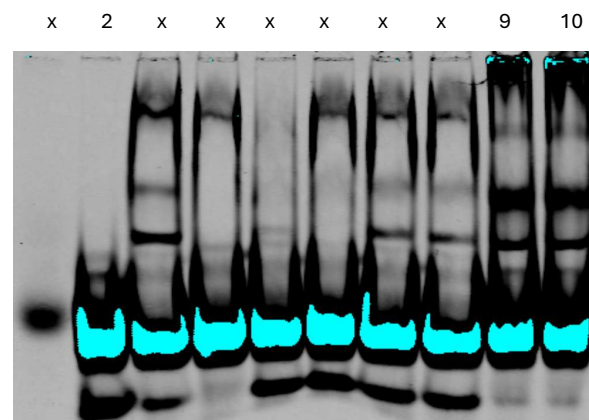

C

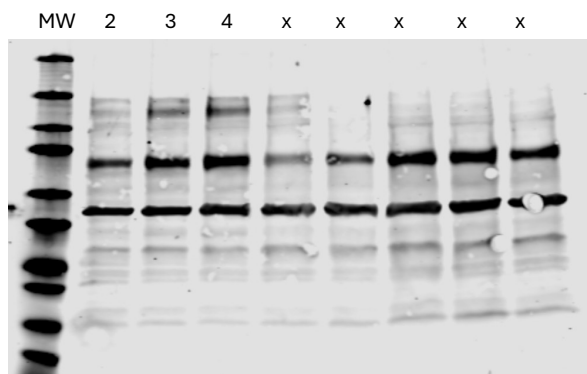

F

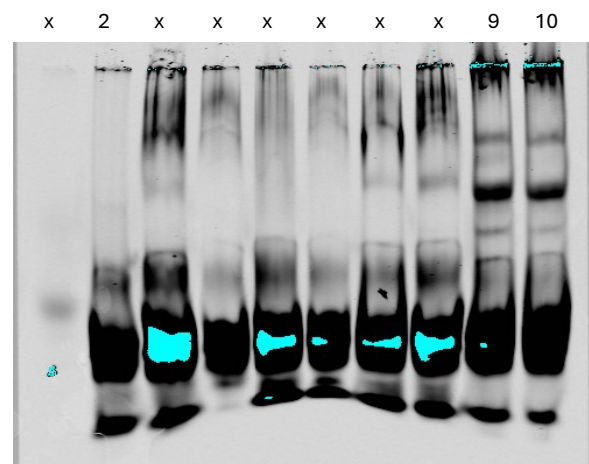

G

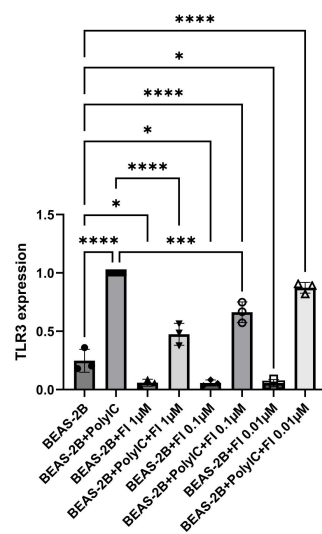

Supplement: S1 Fig — A) Raw Western blot data corresponding to Fig 1B. B) Raw Western blot data corresponding to Fig 3C. C) Raw Western blot data corresponding to Fig 4B. D) Raw EMSA data corresponding to Fig 5B. E) Raw EMSA data corresponding to Fig 5C. F) Raw EMSA data corresponding to Fig 5D. G) FOXO1 inhibitor decreases TLR3 mRNA expression in BEAS-2B cell in a dose-dependent fashion. BEAS-2B cells were treated with Poly(I:C) alone or with increasing concentrations of a FOXO1 inhibitor (0.01µM, 0.1 µM and 1.0 µM). Untreated cells and cells treated with Poly(I:C) alone served as controls. TLR3 mRNA expression was quantified by RT-qPCR and normalized to GAPDH. BEAS-2B cells showed reduced TLR3 expression at baseline compared to cells treated with FOXO1 inhibitor AS1842856. BEAS-2B in the presence of Poly(I:C) and FOXO1 inhibitor AS1842856 showed a dose-dependent reduction in TLR3 mRNA expression (n = 3). (PDF) [file pone.0345169.s001.pdf]
