# Supplementary material for: FOXO1 transcription factor modulates airway epithelial responses to viral infection
Source: PLoS One. 2026 Apr 3;21(4):e0345169. doi: 10.1371/journal.pone.0345169 (PMC13048417; doi:10.1371/journal.pone.0345169)
Supplement: Attachment — Submitted filename: Rebuttal Plos One 22.11.24.docx [file pone.0345169.s003.docx]

Date: 24 November 2025

Manuscript Number: PONE-D-25-41597] - [EMID:191f4936a3b0eb60]

Title of Article: *FOXO1 transcription factor regulates Toll-like-receptor 3 mRNA and coordinates antiviral responses in BEAS-2B airway epithelial cells.*

Name of the Corresponding Author: Nadia Daniel

Email Address of the Corresponding Author: ndaniel@ualberta.ca

Dear Editors,

We thank you for your insightful feedback and comments, and for giving us the opportunity to improve our manuscript.

We have carefully considered each of the comments and made the appropriate changes to our manuscript. We summarize our changes as follows:

- We added additional data and 2 co-authors
- We updated the methodology and the discussion has been extensively reorganized
- We refined the title and text to more accurately reflect the scope of our findings.

We also provide detailed responses to each of the comments and the lines in the manuscript that were revised.

We are happy to answer any further questions and look forward to hearing from you.

Yours sincerely,

Nadia Daniel and Harissios Vliagoftis

**Editor Comments:**

**Comment E1**. Please ensure that your manuscript meets PLOS ONE's style requirements, including those for file naming. The PLOS ONE style templates can be found at

<https://journals.plos.org/plosone/s/file?id=wjVg/PLOSOne_formatting_sample_main_body.pdf> and <https://journals.plos.org/plosone/s/file?id=ba62/PLOSOne_formatting_sample_title_authors_affiliations.pdf>

**Response E1:** The manuscript and associated files have been revised to comply with the PLOS ONE style requirements. We have carefully cross-checked the submission against the PLOS ONE formatting templates provided and confirm that the revised version meets these requirements.

**Comment E2.** PLOS ONE now requires that authors provide the original uncropped and unadjusted images underlying all blot or gel results reported in a submission’s figures or Supporting Information files. This policy and the journal’s other requirements for blot/gel reporting and figure preparation are described in detail at <https://journals.plos.org/plosone/s/figures#loc-blot-and-gel-reporting-requirements> and <https://journals.plos.org/plosone/s/figures#loc-preparing-figures-from-image-files>. When you submit your revised manuscript, please ensure that your figures adhere fully to these guidelines and provide the original underlying images for all blot or gel data reported in your submission. See the following link for instructions on providing the original image data: <https://journals.plos.org/plosone/s/figures#loc-original-images-for-blots-and-gels>.

In your cover letter, please note whether your blot/gel image data are in Supporting Information or posted at a public data repository, provide the repository URL if relevant, and provide specific details as to which raw blot/gel images, if any, are not available. Email us at [plosone@plos.org](mailto:plosone@plos.org) if you have any questions.

**Response E2:** We have provided the original, uncropped, and unadjusted blot images corresponding to all Western blot and EMSA data presented in the manuscript. These raw image files are included in the revised submission as Supporting Information. Each file is labelled to indicate the corresponding figure panel in the main text. All blots included in the manuscript are available, and no raw blot/gel images are missing. In our cover letter, we have confirmed that the original blot image data are provided in the Supporting Information.

**Comment E3.** Please expand the acronym “NSERC” (as indicated in your financial disclosure) so that it states the name of your funders in full. This information should be included in your cover letter; we will change the online submission form on your behalf.

**Response E3:** In our financial disclosure, we have expanded the acronym “NSERC” to its full form: Natural Sciences and Engineering Research Council of Canada (NSERC). This information is also included in the cover letter for clarity.

**Comment E4.** Thank you for stating the following financial disclosure: “This work was supported by a Discovery grant from NSERC and the GSK/CIHR Chair in Airway Inflammation to HV. NMD was supported by an NSERC Canada Graduate Scholarship Doctoral Program studentship. PF is the AstraZeneca (Canada) Inc., Chair in Asthma and Obstructive Lung Disease.” Please state what role the funders took in the study. If the funders had no role, please state: "The funders had no role in study design, data collection and analysis, decision to publish, or preparation of the manuscript." If this statement is not correct you must amend it as needed. Please include this amended Role of Funder statement in your cover letter; we will change the online submission form on your behalf.

**Response E4:**  The funders had no role in study design, data collection and analysis, decision to publish, or preparation of the manuscript. We have added this statement to the cover letter as requested.

**Comment E5.** Thank you for stating the following in the Acknowledgments Section of your manuscript: “This work was supported by a Discovery grant from NSERC and the GSK/CIHR Chair in Airway Inflammation to HV. NMD was supported by an NSERC Canada Graduate Scholarship Doctoral Program studentship. PF is the AstraZeneca (Canada) Inc., Chair in Asthma and Obstructive Lung Disease. We would want to acknowledge Dr. Fred Berry for his help with the EMSA, and Luke Gerla and Marc Duchenne for their help with cytokine measurements.”

We note that you have provided funding information that is not currently declared in your Funding Statement. However, funding information should not appear in the Acknowledgments section or other areas of your manuscript. We will only publish funding information present in the Funding Statement section of the online submission form.
Please remove any funding-related text from the manuscript and let us know how you would like to update your Funding Statement. Currently, your Funding Statement reads as follows:
This work was supported by a Discovery grant from NSERC and the GSK/CIHR Chair in Airway Inflammation to HV. NMD was supported by an NSERC Canada Graduate Scholarship Doctoral Program studentship. PF is the AstraZeneca (Canada) Inc., Chair in Asthma and Obstructive Lung Disease. Please include your amended statements within your cover letter; we will change the online submission form on your behalf.

**Response to Comment E5:** All funding-related text has been removed from the Acknowledgments section of the manuscript, leaving only acknowledgment of individual contributions. The corrected Funding Statement is provided in the cover letter, as requested:

**Comment E6.** We notice that your supplementary figures are uploaded with the file type 'Figure'. Please amend the file type to 'Supporting Information'. Please ensure that each Supporting Information file has a legend listed in the manuscript after the references list.

**Response E6:** All supplementary figure files have been re-uploaded under the file type “Supporting Information,” and each is now provided with a corresponding legend listed in the manuscript after the References section, as required.

**Comment E7.** If the reviewer comments include a recommendation to cite specific previously published works, please review and evaluate these publications to determine whether they are relevant and should be cited. There is no requirement to cite these works unless the editor has indicated otherwise.

**Response E7:** We carefully reviewed all of the publications suggested by the reviewers and have cited those we deemed relevant and appropriate in the revised manuscript.

**Comment R1.1:** The authors claim that FOXO1 regulation of TLR3 in airway epithelial cells is important for inflammatory cytokine production in antiviral innate immunity. However, TLR3 was evaluated only at the mRNA level (Figure 3). Although the changes may be statistically significant, the biological impact is unclear. It is essential to examine whether TLR3 protein levels are also reduced, as mRNA changes do not necessarily correspond to protein changes. Since TLR3 immunoblotting is shown in Figure 4, such experiments should be feasible.

**Response R1.1:** We performed western blot analysis of TLR3 protein following FOXO1 knockdown. These experiments showed no statistically significant differences in TLR3 protein expression compared with scrambled controls, despite the consistent reduction at the mRNA level. We have now included these data in the revised manuscript (new Figure 3C&D). We agree that reduced TLR3 mRNA without detectable protein change may limit the functional impact of our observation, and we have expanded on this point in the revised Discussion (lines 428-431).

**Comment R1.2:** While the authors discuss the importance of TLR3 in antiviral innate immune responses in airway epithelial cells, it is well established that in non-professional immune cells, viral RNA sensing is primarily mediated by RIG-like receptors such as RIG-I, whereas TLR3 serves as a major dsRNA sensor in professional immune cells like dendritic cells. The study does not address the contribution of RIG-I at all. Although the focus on TLR3 is understandable, the authors should also investigate the effect of FOXO1 knockdown/overexpression on RIG-I expression and signaling.

**Comment R1.3:** The regulation of TLR3 expression by FOXO1 may be a secondary effect; the role of FOXO1 downstream of RIG-I–MAVS or TLR3–MyD88 signaling may be more important. Prior studies (PMID: 30944148) have shown that FOXO1 is involved in controlling TLR3-dependent cytokine expression. There are also reports on FOXO1 regulation in RIG-I signaling (PMID: 33187908). If the authors aim to emphasize the importance of FOXO1-mediated regulation of TLR3, they should also present data on FOXO1 function in TLR3 and RIG-I pathways.

**Response R1.2 & R1.3:** We agree with the reviewers that other antiviral pathways are also very important in airway epithelial cells. To this effect, we performed qPCR analyses of DDX58 (RIG-I) and its signaling adaptor MAVS, as well as MYD88, in FOXO1 knockdown BEAS-2B cells at baseline and after poly(I:C) stimulation (8 h and 24 h). We observed no significant changes in expression levels of RIG-I, MAVS, or MYD88 between FOXO1 knockdown and control cells under these conditions.

We recognize that prior studies have reported roles for FOXO1 in antiviral signaling. PMID: 30944148 showed that in human mesenchymal stromal cells, FOXO1 regulates TLR3-dependent cytokine and migration responses, whereas PMID: 33187908 demonstrated that FOXO1 negatively regulates RIG-I signaling by limiting TRAF3 ubiquitination and IRF3 stability, thereby reducing type I IFN production. In airway epithelial cells, FOXO1 knockdown decreases TLR3 mRNA but not protein, so we do not infer regulation of receptor abundance. Instead, our findings suggest that FOXO1 primarily modulates downstream signaling competence in response to viral RNA sensors, consistent with its signaling roles reported in other systems. This likely reflects pathway-dependent regulation, where FOXO1 influences antiviral signaling outputs according to the dominant RNA-sensing mechanisms active in each cell type.

We have added these data as Figure 4F+G+H in the revised manuscript. We agree that the absence of changes in RIG-I, MAVS, or MYD88 suggests that FOXO1’s regulatory effects are selective rather than global, and we have revised the Discussion to reflect this specificity (lines 468-473).

**Comment R1.4:** On line 300, the authors state that FOXO1 is essential for the constitutive expression of TLR3. However, well-known TLR3-dependent genes such as IP-10 and IL-8, which have been reported to be induced by poly(I:C), show no changes according to Figure S1. This is a major inconsistency that requires clarification.

**Response R1.4:**  As we also mentioned above, although FOXO1 knockdown consistently decreased TLR3 transcript levels, this was not accompanied by detectable differences in TLR3 protein or in the induction of these canonical TLR3-dependent cytokines. We have therefore revised the text to remove the statement that FOXO1 is “essential” for constitutive TLR3 expression and now state that FOXO1 influences TLR3 transcription, but the biological significance of this change remains unresolved (lines 428-429). This probably explains why both baseline and poly(I:C)-activated IP-10 and IL8 expression is not altered in FOXO1 deficient cells.

**Comment R1.5:** While FOXO1 appears to suppress IL-6 and CCL2 expression, the effect is limited, and for IL-6 it explains only part of the observed changes. This point requires further discussion or additional experimental validation.

**Response R1.5:** We agree that FOXO1’s regulatory effect on IL6 and CCL2 is partial. These results are, however, consistent with previous reports for FOXO1-dependent regulation of these two cytokines by TLR3 in mesenchymal stromal cells and adipocytes (Kim et al.,2019 and Ito et al.,2009). In addition, both IL6 and CCL2 are well recognized to be under the control of multiple transcriptional regulators, including NF-κB, MAPKs, STATs, and IRFs, which act in parallel or redundant fashion to amplify inflammatory outputs. Our findings therefore suggest that FOXO1 functions as a modulator rather than a sole regulator of these cytokines in airway epithelial cells. We have revised the Discussion (lines 433-443) to highlight this point and to caution against over-attribution of IL6 and CCL2 regulation to FOXO1 alone.

**Comment R1.6:** The current data are based on airway epithelial cells. Is the observed FOXO1 function specific to airway epithelial cells, or is it a general mechanism across cell types? This point should be discussed.

**Response R1.6:** Our study is focused on airway epithelial cells given their critical role in frontline antiviral defense and the particular interest of our laboratory to lung immunity. However, we agree it is important to present our data in the context of the extensive regulatory role of FOXO1 in other cell types and other tissues. Prior literature demonstrates that FOXO1 participates in immune and inflammatory regulation across diverse systems. For example, FOXO1 regulates macrophage polarization in allergic inflammation (Chung et al., 2016), promotes IRF3 degradation to dampen antiviral responses (Lei et al., 2013), and controls IL6 and CCL2 induction downstream of TLR3 in mesenchymal stromal cells (Kim et al., 2019). FOXO1 has also been shown to drive proinflammatory gene expression in adipocytes (Ito et al., 2009) and to enhance chemokine-mediated lymphocyte recruitment (Miao et al., 2012). More recently, microRNA-dependent regulation of FOXO1 was linked to inflammatory responses in macrophages (Hu et al., 2022). We have expanded the discussion (lines 456-464) to include the studies cited above to help the reader appreciate the wider context of the FOXO1 effects on the immune system.

**Comment R1.7:** Since this is presented as a study of antiviral responses in airway epithelial cells, at least one set of data from an actual viral infection experiment should be provided.

**Response R1.7:** We agree with the reviewer that data on the role of FOXO1 in viral infections of airway epithelial cells would strengthen our findings. To address this, we performed an infection experiment in primary human airway epithelial cells (NHBE). Cells were infected with SARS-CoV-2 VIDO-01 (MOI = 0.5) in the presence or absence of a FOXO1 inhibitor, and total RNA was harvested at 24 h post-infection. Viral RNA levels were quantified by qRT-PCR (spike mRNA normalized to β-actin; *n* = 3; paired *t*-test). FOXO1 inhibition significantly reduced spike mRNA relative to DMSO controls (Figure 4I). These results and discussion (lines 350-353 & 474-484) are now updated in the revised manuscript and further highlight the context-dependent role of FOXO1 in modulating TLR3-dependent signaling in airway epithelial cells.

**Minor Point 1:** Figure 1F is too dark to clearly visualize FOXO1-positive staining; a brighter image should be provided.

**Response:** Figure 1F has been replaced with a brighter image to allow clearer visualization of FOXO1-positive staining.

**Minor Point 2**: Figure S1 presents important findings and should be moved from the supplemental data to the main figures.

**Response:** We appreciate the reviewer’s suggestion. Figure S1 has been moved from the supplemental data to the main figures (now presented as Figure 3 in the revised manuscript).

**Minor Point 3**: Likewise, the results in Figure S4 should be presented as a main figure rather than in the supplemental section.

**Response:** Figure S4 has been incorporated into the main figures (now presented as Figure 5 in the revised manuscript).

**Comment R2.1:** Please justify and confirm use of 50ug/ul Poly (I:C) concentration for TLR3 agonism (line 246 states “based on these findings” 50ug/mL was used, but only one concentration was tested, and this was different from the methods where 50ug/ul was stated).

**Response:** We thank the reviewer for pointing out the typo in our Methods section and for requesting clarification on Poly(I:C) dosing. The unit has been corrected from “50 µg/µL” to 50 µg/mL in the revised manuscript and the statement has been updated (line 311).

**Comment R2.2:** It is not clear in the text how FOXO1 is suggested to become active and translocate to the nucleus, which would justify why the constitutively active FOXO1 mutant (CA-FOXO1) was used. It seems that this mutant is phosphorylation-deficient, meaning phosphorylation inhibits its activity, so please describe this in the text.

**Response:** In the revised manuscript, we have expanded our description of FOXO1 regulation and clarified the rationale for using the constitutively active FOXO1 (CA-FOXO1) construct. FOXO1 activity is normally controlled through post-translational modifications, most prominently phosphorylation by the PI3K–Akt pathway. Phosphorylation at Thr24, Ser256, and Ser319 drives FOXO1 nuclear export and degradation, thereby suppressing its transcriptional activity. In contrast, the CA-FOXO1 mutant contains alanine substitutions at these phosphorylation sites, rendering it resistant to Akt-mediated inhibition. As a result, CA-FOXO1 remains nuclear and transcriptionally active independent of upstream signaling.

We employed the CA-FOXO1 mutant to test whether enforced nuclear activity of FOXO1 is sufficient to regulate TLR3 expression and downstream responses. This gain-of-function approach complements our loss-of-function experiments (FOXO1 knockdown) and provides a direct means to assess the consequences of persistent FOXO1 activity.

We have revised the Methods and Results sections (lines 111-114 and 337-343) to incorporate these details and to emphasize how CA-FOXO1 differs from endogenous FOXO1 in terms of phosphorylation control and activity.

**Comment R2.3**: In the methods, please specify that nuclear extracts were used for EMSA and how these were prepared, in S4 it is stated nuclear extracts were used but this was not in the methods. Please also clarify all the components that were used in the “EMSA buffer”, or if this was part of commercial kit.

**Response:** We appreciate this suggestion. In the revised manuscript, we have added full methodological details for our EMSA experiments (lines 204-218).

**Comment R2.4:** (Figure 1) For the FOXO1 shRNA transduced cells, the western blot shows overall less FOXO1 compared to control, but this is much less clear in the immunofluorescence data where only a drop in nuclear localization is found. Please show whole cell fluorescent intensity for FOXO1 as well.

**Response:** We thank the reviewer for this valuable suggestion. In the revised figures, we now include quantitative analysis of whole-cell FOXO1 fluorescence intensity in BEAS-2B cells following shRNA-mediated FOXO1 knockdown, in addition to the nuclear localization data previously shown. Whole-cell fluorescence intensity was quantified using Volocity Analysis software, with phalloidin staining used to define cell outlines, and mean FOXO1 fluorescence intensity was calculated per cell. This analysis confirms that FOXO1 knockdown reduces overall FOXO1 protein levels, consistent with the western blot data. By presenting both nuclear localization and overall fluorescence intensity, the data more clearly demonstrate the effectiveness of FOXO1 knockdown and align the immunofluorescence findings with the immunoblotting results. We have updated Figure 1F and its legend to include these quantifications.

**Comment R2.5:** (Figure 1) It would be useful to show reduction in TLR3 protein (ie. through western blot since you have a working antibody) as well as the mRNA in Figure 1 following FOXO1 shRNA, since this links the regulatory element of your story to the functional statements in Figure 2.

**Response:** Please see also our response to R1.1 for Figure 3. In brief, in the revised manuscript we suggest that FOXO1 shRNA decreases TLR3 mRNA expression, but not the expression of TLR3 protein.

**Comment R2.6:** (Figure 4) It is not clear what the significance of TLR3 regulation by FOXO1 is, if TLR3 expression is not impacted at the protein level, and how this contributed to the functional differences you observe? Since it is stated that (potentially) a different timepoint could be used to capture protein changes, this should be done tested, or ensure that the antibody is detecting the correct target. The same goes for figure 3 with the FOXO1 inhibitor.

**Response:** The same concern regarding the relationship between TLR3 mRNA and protein levels, and the timing of potential protein changes, was raised by Reviewer 1 and is addressed in Response R1.1. These issues are discussed in lines 428-431 of the manuscript**.**

**Comment R2.7:** (Figure 5) Please describe whether these ChIP datasets used are treated or use constitutively active FOXO1. It is not clear whether there is any major binding above the background signal at the two FOXO1 motifs selected, likely because there is not much FOXO1 binding to the TLR3 locus without simulation. Therefore, this alone can not be used as evidence that FOXO1 can bind the TLR3 locus in any cell type. It is suggested to perform a ChIP-PCR in Poly-IC stimulated conditions (where there would be greater binding compared to background) or find a different data set that is more convincing.

**Response:** The ChIP datasets we utilized were derived from untreated cells expressing constitutively active FOXO1. Importantly, the binding signals observed at the TLR3 locus are statistically significant and exceed background levels, as indicated by the ChIP-Atlas analysis. These regions were not selected based on the presence of FOXO1 motifs in the DNA sequence; rather, they were identified through unbiased analysis of FOXO1 binding peaks. While we acknowledge that stimulation (e.g., with Poly-IC) may enhance FOXO1 binding at this locus, the current data provide evidence of basal-level interaction in the context of constitutively active FOXO1. We have clarified this point in the revised manuscript (lines 382-389) to better reflect the nature of the datasets and the interpretation of the binding signals.

**Comment R2.8:** (Figure S5) Although there is evidence of some binding of proteins within the nuclear extract to the oligo in the EMSA based on lane 2 compared to lane 3 (panel A, labelled “complex 2”), all other labelled complexes are present in the probe only lane so these should not be discussed as binding in panel B+C. Additionally, this probe only control should also be present in panels B+C. Since “complex 2” does not super-shift with FOXO1 antibody, there is no evidence that FOXO1 binds this oligo, even in constitutively active conditions. There is quite a lot of DNA on either end of the 4bp FOXO1 binding site, so it could be a host of other factors binding to this oligo, and this is not in the native chromatin conformation or cell environment that ChIP-PCR would allow.

**Response:** We have revised the presentation of the data from our EMSA analysis to clearly note in the *Results* that several bands are also present in the probe-only control, indicating non-specific binding. Figure 5 has been updated to include probe-only controls in panels B and C. We also clarify that our original text did not claim definitive FOXO1 binding and have revised the *Results* and *Discussion* (lines 390-401 & 504-511) to emphasize that the EMSA data are inconclusive and should not be interpreted as evidence of direct FOXO1 occupancy.

**Comment R2.9:** The “signalling” part of the title should be removed unless additionally evidence is provided that TLR3 signalling, ie. through phospho-IRF3, phospho-IKKε, phospho-TBK1, or ISG activity is impacted by FOXO1 depletion/inhibition (and that TLR3 protein is reduced by FOXO1 depletion as stated above). Instead focus on the barrier function and cytokine expression impacts of FOXO1.

**Response:** We agree that the evidence currently presented does not conclusively demonstrate altered TLR3 signaling since we have not tested any downstream phosphorylation events (e.g., phospho-IRF3, phospho-TBK1, phospho-IKKε) or analyzed any signaling pathways. We believe that it is likely that the changes seen in cytokine/chemokine production following TLR3 activation in FOXO1 deficient cells are the result of signalling events, since there is no change in TLR3 protein expression. However, given the absence of direct signaling readouts, we have revised the title to *“FOXO1 transcription factor regulates Toll-like-receptor 3 mRNA and coordinates antiviral responses in BEAS-2B airway epithelial cells.”*

**Review Question:** Is the manuscript presented in an intelligible fashion and written in standard English?

Answer: Party (only yes or no option available)

Story is overall intelligible, but some changes are suggested. Some grammatical errors are not noted here so please review text before resubmitting.

Line 206 grammar “of by total cell counts at the end of incubation”

Line 206 “Barrier integrity showed that both FOXO1-deficient and scrambled shRNA”, explain how resistance relates to barrier integrity ie. higher ohms tighter barrier: barrier integrity did not “show”, higher olms indicated increased barrier integrity

Line 228 “FOXO1 downregulation did not affect baseline release of cytokines from BEAS-2B cells” unknown whether this is all cytokines, only chosen/select cytokines

Line 242 Specify activation is by poly-IC in paper cited, would help with overall story

Line 281 Please indicate whether this data is or is not shown

**Response:** We have carefully revised the manuscript for clarity and grammar to improve readability and ensure that the text is written in standard English. All sections have been re-checked and edited to address grammatical errors and to enhance overall flow.
