# Supplementary material for: FOXO1 transcription factor modulates airway epithelial responses to viral infection
Source: PLoS One. 2026 Apr 3;21(4):e0345169. doi: 10.1371/journal.pone.0345169 (PMC13048417; doi:10.1371/journal.pone.0345169)
Supplement: Attachment — Submitted filename: Response to Reviewers letter 20260217.docx [file pone.0345169.s004.docx]

**Rebuttal Letter – Full Revised Version (Round 2) - 20260217**

**Revised Title:** *“*FOXO1 transcription factor modulates airway epithelial responses to viral infection*”*

**Manuscript ID:** PONE-D-25-41597R1	**Journal:** PLOS ONE

**Reviewer #1 indicated that their concerns were fully addressed.**

**Reviewer #2 Comment: *“****While I appreciate the inclusion of TLR3 protein data, the manuscript needs to address more clearly how FOXO1 would modulate TLR3 signalling (or signalling via other viral PRRs/RLRs) if this modulation of transcription is not apparent at the protein level. If TLR3 expression was altered, the change in title would have been sufficient, however due to this null finding I still would recommend that additional experiments are completed to explore other ways in which FOXO1 could modulate cytokine release and viral signalling (ie. through regulation of other levels of PRR signalling p-TBK1, p-IRF3, NF-κB activation etc.), since the claims about TLR3 mRNA regulation, repressed cytokine release, as well as viral modulation are currently disconnected. Alternatively, claims about viral modulation via FOX01 could be isolated to a different manuscript, and this paper could focus solely on the regulation on TLR3 mRNA by FOXO1, however a more definitive EMSA or alternative way to show FOXO1 binding to DNA in airway epithelial cell lines would be required. As the manuscript currently stands I don’t believe “conclusions are presented in an appropriate fashion and are supported by the data”, as per the PLOS One publication standards, since the title implies that FOX01 coordinates antiviral responses through TLR3 dependent mechanism, and there is a lack of evidence for this.”*

**Response to Reviewer #2:**

We understand the reservations of the reviewer regarding the role of FOXO1 in TLR3 signaling. To support the conclusion that FOXO1 participates in the epithelial response to TLR3 activation, we performed immunofluorescence experiments demonstrating increased FOXO1 nuclear localization following Poly(I:C) stimulation **(Fig. 5A+B).** We therefore propose that altered outcomes of TLR3 activation (i.e. increased TLR3 mRNA expression and increased release of certain cytokines) in the absence of FOXO1 are most likely the result of lack of TLR3-induced FOXO1 activation and probably not the result of altered TLR3 expression. We do not claim definitive FOXO1 control of canonical downstream signaling pathways or direct binding to the TLR3 promoter.

Unfortunately, further studies on the effects of FOXO1 downregulation on signaling pathways activated by TLR3 are difficult to execute at this time, as we do not yet know which specific pathways are affected or whether FOXO1 acts upstream or downstream of other proteins in these pathways. Identifying these pathways is a major component of our future plans for this project, but we feel that pursuing this level of pathway dissection would be beyond the scope of the current manuscript.

In addition, FOXO1 deficiency alters epithelial barrier properties, suggesting a mechanism by which FOXO1 may influence host–virus interactions at the epithelial surface. Disruption of epithelial barrier properties can affect viral access to underlying immune compartments and modulate local inflammatory responses. In line with this epithelial-focused role, our SARS-CoV-2 data indicate that FOXO1 participates in epithelial responses activated during viral infection. Together, these observations support a model in which FOXO1 selectively shapes epithelial inflammatory responses through effects on both transcriptional programs and barrier properties, rather than acting as a global regulator of antiviral signaling.

To clarify the distinction between FOXO1’s effects on TLR3 expression and its effects on downstream TLR3-activated pathways as well as other anti-viral pathways, we reorganized the Results and Discussion sections to improve clarity. We believe that our conclusions are now supported by the data.

Following the reviewer’s guidance and to better align the title with the data presented, we propose revising the manuscript title from “FOXO1 transcription factor regulates Toll-like receptor 3 mRNA and coordinates antiviral responses in BEAS-2B airway epithelial cells” to “**FOXO1 transcription factor modulates airway epithelial responses to viral infection.***”*

Thank you for your time and consideration.

Sincerely,

Nadia M. Daniel, MSc
(on behalf of all authors)
